# An Improved Peer-Review System to Compensate for Scientific Misconduct in Health-Sensitive Topics

**DOI:** 10.3389/phrs.2023.1605601

**Published:** 2023-06-02

**Authors:** Alessandro Rovetta, Rossana Garavaglia, Alessandro Vitale, Ettore Meccia, Behailu Terefe Tesfaye, Paolo Mezzana, Vincenzo Accurso

**Affiliations:** ^1^ R&C Research, Bovezzo, Italy; ^2^ Fondazione Achille Sclavo Onlus, Siena, Italy; ^3^ Department of Surgical, Oncological and Gastroenterological Sciences (DiSCOG), Padova University, Padova, Italy; ^4^ Istituto Superiore di Sanità, Rome, Italy; ^5^ Clinical Pharmacy Unit, School of Pharmacy, Jimma University, Jimma, Ethiopia; ^6^ Delle Medical Center, Rome, Italy; ^7^ Polyclinic University Hospital, Palermo, Italy

**Keywords:** public health, ethics education in medicine and public health, peer review crisis, BIV1-CovIran, misconduct

## Introduction

In December 2021, one of the authors of the present paper (AR) took part in the peer review of the paper “*Safety and immunogenicity of an inactivated virus particle vaccine for SARS-CoV-2, BIV1-CovIran: findings from double-blind, randomized, placebo-controlled, phase I and II clinical trials among healthy adults*” for the BMJ Open [1, 2]. The manuscript described clinical phases I and II of the COVID-19 vaccine BIV1-CovIran by Shifa Pharmed Industrial Group. The article was accepted for publication in March 2021 after three review rounds, with a total of six reviewers involved. On May 2022, AR received an email from Yeganeh Torbati, a Washington Post reporter who was investigating the development of BIV1-CovIran. Torbati asked AR for a general opinion about the data presented in the above article. AR replied that no serious anomalies were highlighted, although he specified that the peer review process was too superficial to guarantee complete integrity. Subsequently, through an article published in the Washington Post in August 2022, Torbati disclosed serious misconduct dynamics [3]. In support of her claims, an official correction was published in the BMJ Open in November 2022, in which the authors were forced to admit various conflicts of interest and the occurrence of vaccine-related adverse effects [1]. The relevant fact is that not even six peer reviewers and one editor have discovered such a hidden scenario. This is not intended to blame the journal or the reviewers but only to denounce that the world of scientific publication is currently subject to easy ethical violations. Although financial relationships can markedly bias biomedical research, marginal importance is given to this aspect [4, 5]. In this regard, this letter proposes a set of practices to counteract some major integrity problems.

## What Can Authors Do?

*A1*. Authors should facilitate research reproducibility to boost peer-review speed and accuracy. This includes 1) sharing codes, calculations, and data (raw and elaborated), and 2) providing a step-by-step description of the ideas that led to the realization of the project, the implementation of the methods, and the procedures to assess the tests’ assumptions.

*A2.* Authors should adopt frameworks for enhancing quality in preclinical data since this can significantly increase transparency and trust in results and allow errors to be prevented rather than detected too late [6].

*A3*. Regarding clinical trials, authors should publicly share audit/monitoring documents, information about the contract research organization that monitored the study, and data submitted to regulatory agencies (at least on the clinical testing front, which does not seem to threaten intellectual property or industry secrets).

*A4*. Authors should release a preprint version so as to allow the scientific community to review the results independently and rapidly.

## What Can Academic Journals Do?

*J1*. Journal editors should evaluate the paper’s health sensitivity and decide whether it is a high-sensitivity topic. All research involving novel drugs, vaccines, and therapeutic strategies should be considered at high sensitivity.

*J2*. For high-sensitivity topics, journals should compulsorily require *A1–A4*. Any draft version that has passed peer review should be released at the very moment of approval, explicitly indicating that it is an unedited peer-reviewed version. Reviewers’ reports and authors’ responses should always be published, ensuring easy citability (e.g., DOI). Reviewers’ names and affiliations should also be published unless they express reasonable fears for their safety. This should help reduce the problem of coercive citations [7]. Finally, journals should allow authors to publicly share editorial rejection decisions, including reviewers’ reports.

*J3.* For high-sensitivity topics, journal editors should stratify the peer review to ensure the validity of the key elements. Alongside a general assessment, each methodological aspect (e.g., design, population, data collection, statistical analysis, and results) should be carefully evaluated by one or more independent specific experts, especially when dealing with high-complexity data or multidisciplinary approaches. Regarding clinical trials, editors should involve expert figures to evaluate pharmacological and public health aspects (e.g., adverse reaction reports). Journals should also include a specific mandatory section in which reviewers declare the limitations of their review (e.g., “I’m not an expert on Bayesian methods”), so that editors and readers have a clear understanding of what the reviewers assessed. Finally, double-blind review should be required to reduce authorship bias [8].

*J4*. For high-sensitivity topics, journal editors should create a dedicated section made up of two or more journalists experienced in detecting ethical violations. Such supervision should extend to the authors but also the reviewers, who could voluntarily influence the publication process. The inquiry must only concern researchers’ professional relationships and activities, without affecting the private sphere, in order to safeguard their privacy. The academic journal should propose to the reporters to sign a non-disclosure agreement regarding the data found and guarantee the quality of the investigation.

*J5*. For high-sensitivity topics, journals should compulsorily require that the data are suitably standardized to allow decentralized analysis through automated tools, software, or artificial intelligence algorithms [6, 9]. Specific guidelines should be provided to help authors with the A1 point. Means for decentralized analysis should be provided to reviewers. Should a unique international standardization be chosen by regulatory agencies, journals would have to adhere to it.

*J6*. For high-sensitivity topics, journals should pay peer reviewers and editors. Indeed, paying peer reviewers—a sustainable practice, as shown by the editorial policies of various journals—would foster excellence thanks to an economic reward proportional to the reviewer’s skill (competition mechanism). One of the main obstacles to publication, namely, the difficulty in finding available reviewers, would be quickly overcome. Scientists could play this role on a permanent and ongoing basis thanks to the benefits of true job performance. Paid work would increase the actual responsibility of peer reviewers and editors.

## What Can Abstracting Service Groups Do?

*I1*. Tiered indexing should be introduced by abstracting services. The top rank should only be granted to academic journals that meet *J1–J6*. Indeed, since indexing in recognized databases is a source of prestige (so much so that, in most cases, journals reserve a special section of their websites to this scope), doing so would drive health journals to adjust to the new standards. Moreover, this would help the public to identify the most authoritative and reliable journals. Similar initiatives are already underway [10].

## What Can Regulatory Agencies, Funders, and Institutions Do?

*R1*. Funders and regulatory agencies should require necessary authors’ compliance with points *A1*–*A3*.

*R2.* Regulatory agencies should agree on a unique international data standardization (see point *J5*) so as to strengthen and accelerate scrutiny by the whole scientific community.

*R3*. Institutions and employers should actively encourage and support scientific refereeing. Moreover, funders should be willing to finance an extra amount to properly perform points J3, J4, and J6.

In conclusion, we do ask the scientific community to take a clear position and make itself heard with a stentorian voice to protect public health from ethical misconduct. This renewal would lead not only to direct benefits to the research but also to the public image of the whole scientific world, thanks to a novel, more transparent, efficient, and effective procedure of academic publication. We are aware that these guidelines are tailored to the medical field and that some of our requests could be not applicable or not stringent enough. Therefore, if needed, specific recommendations should be added or lifted based on the research field.
